# Assessing the Influence of Long-Term Gender-Affirming Hormone Therapy on Cardiovascular Risk in Transgender Men through Carotid Intima–Media Thickness

**DOI:** 10.3390/jcm13196001

**Published:** 2024-10-09

**Authors:** Rauf Hamid, Abdulkadir Güllüce, Osman A. Kargın, Seyfullah H. Karagöz, İbrahim Adaletli, İsmail Çepni, Abdullah Tüten

**Affiliations:** 1 Department of Radiology, Cerrahpasa School of Medicine, Istanbul University-Cerrahpasa, Istanbul TR-34320, Turkey; drraufhamid@hotmail.com (R.H.); seyfullahhalitkaragoz@gmail.com (S.H.K.); ibrahim.adaletli@iuc.edu.tr (İ.A.); 2 Department of Obstetrics and Gynecology, Cerrahpasa School of Medicine, Istanbul University-Cerrahpasa, Istanbul TR-34320, Turkey; kadirgulluce@gmail.com (A.G.); ismailcepni@gmail.com (İ.Ç.); abdullah.tuten@istanbul.edu.tr (A.T.); 3 Department of Radiology, İstanbul Physical Therapy and Rehabilitation Training and Research Hospital, University of Health Sciences, Istanbul TR-34182, Turkey

**Keywords:** carotid intima-media thickness, transgender men, gender-affirming hormone therapy

## Abstract

**Background:** Transgender men use exogenous androgen for male pattern virilization. Hysterectomy and bilateral salpingo-oophorectomy (HBSO) is performed to stop the endogenous estrogen secretion. Cardiovascular disease (CVD) risk has been shown to increase with long-term use of androgens and the removal of estrogen. We aimed to investigate the CVD risk in these individuals by measuring internal and common carotid artery intima–media thicknesses (CIMT). **Methods:** In this cohort study, data were collected from transgender men who had undergone HBSO and used androgens for at least two years (median treatment duration was 5 years in our research). Cisgender women in the same age range were selected as the control group. Demographics, vital signs, and hematological values of transgender patients and cisgender women subjects in the control group were noted. CVD markers were compared with sonographically measured CIMT values. **Results:** The mean age and body mass index (BMI) of the study group were 32.6 and 25.3, respectively. Weight, systolic–diastolic blood pressure, hemoglobin, hematocrit, low-density lipoprotein (LDL), serum triglyceride (TG), HbA1c levels, internal CIMT, and common CIMT values of the study group were higher, while the high-density lipoprotein (HDL) level was significantly lower compared the control group (p1 = 0.025, p2 = 0.010, p3 = 0.002, p4 = 0.001, p5 = 0.001, p6 = 0.012, p7 = 0.008, p8 = 0.007, p9 = 0.013, and p10 = 0.001). There was also an increase in the body weight, BMI, LDL, and TG levels of the study group after the testosterone treatment (p1 = 0.025, p2 = 0.019, p3 = 0.001, p4 = 0.001, and p5 = 0.001). **Conclusions:** We demonstrated that the use of testosterone therapy in transgender men is associated with higher CIMT values. While further investigation is needed to assess morbidity and mortality rates, we recommend that regular clinical and radiological examinations be performed in these individuals to accurately evaluate the risk of CVD.

## 1. Introduction

Gender dysphoria is a condition characterized by a discrepancy between an individual’s gender identity and their assigned sex at birth, encompassing persistent identification with the opposite sex and a desire to be recognized as a member of that gender [1]. While the true prevalence of transgender individuals is unknown, the increasing demand for healthcare services among this population underscores the growing need for our engagement. Understanding their unique health risks and needs will contribute to improving overall public health.

The primary objective of long-term gender-affirming hormone therapy in transgender men is to induce virilization, encompassing various outcomes such as the cessation of menstruation, the development of masculine physical characteristics, deepening of voice, increased sexual desire, the growth of facial and body hair, and clitoral hypertrophy [2,3,4]. Testosterone is the main agent used to achieve these effects [2,5]. While various testosterone hormone formulations are available in different geographical regions, testosterone esters are generally utilized. Studies have indicated no significant difference in drug efficacy or side effects among different forms of these testosterone esters, including oral, transdermal, and parenteral solutions [6,7]. Hormonal therapy is typically recommended for life-long use to prevent the development of vasomotor symptoms associated with hypogonadism and to prevent osteoporosis [8].

In recent decades, the adverse effects of androgens on the cardiovascular system have gained prominence as an important topic of concern. Adult men have a higher risk of developing cardiovascular disease than women until menopause, primarily due to the differential impacts of sex hormones [9,10]. In women, conditions that lead to a decrease in circulating estrogens, such as aging or surgery, are known to cause menopausal symptoms like vasomotor attacks, sleep disturbances, depression, and bone loss. Additionally, early menopause, occurring before the age of 40, affects approximately 1% of women and is associated with an elevated risk of cardiovascular mortality [11,12]. Hormone replacement therapy is often employed to alleviate symptoms, and when initiated within the first five years of menopause, it demonstrates cardioprotective benefits [11,13]. Evidence suggests that estrogen exerts antiplatelet, antioxidant, and vasodilatory effects, possibly contributing to cardiovascular protection [14,15]. On the other hand, testosterone is believed to have the opposite effect, potentially increasing the risk of cardiovascular disease.

However, the limited number of studies on transgender men using exogenous testosterone have not shown any adverse cardiovascular events [16,17,18]. Furthermore, both short- and long-term studies have revealed no significant differences in cardiovascular mortality rates between transgender men and control groups [9,10,17,18,19]. Recently published, the TRAVERSE trial argues that in men with hypogonadism and preexisting or a high risk of cardiovascular disease, testosterone replacement therapy was non-inferior to placebo with respect to the incidence of major adverse cardiac events [20]. A cross-sectional study showed no myocardial infarction, stroke, or deep vein thrombosis in 50 transgender men who received testosterone therapy for ten years [21]. In a similar cohort study, a low risk of cardiovascular morbidity was observed in 138 transgender men receiving testosterone ester injections every 3 weeks [22].

In contrast, recent research published in four different papers has demonstrated the adverse effects of testosterone hormone therapy on cardiovascular disease risk factors. These effects include an increase in hemoglobin and hematocrit levels; a decrease in HDL levels; an increase in LDL levels, TG levels, and inflammation parameters; a minimal rise in systolic blood pressure; and a decrease in adiponectin and leptin levels [16,17,23,24]. The variation in the results across different studies examining transgender men undergoing gender-affirming hormone therapy may be attributed to the fact that the majority of these individuals are relatively young, and cardiovascular events are less common in this age group. Therefore, there is a need for either long-term studies involving older transgender individuals or the use of novel sensitive biomarkers capable of detecting cardiovascular event risks at earlier stages.

It is essential to identify asymptomatic patients at a high risk of cardiovascular disease to prevent morbidity and mortality resulting from cardiovascular complications [25]. The screening test employed to detect such patients should be safe, sensitive, and cost effective. The measurement of carotid intima–media thickness (CIMT) with B-mode ultrasound is a non-invasive, sensitive, and reproducible technique for detecting subclinical vascular disease and assessing the risk of cardiovascular complications [26].

**Figure 1:**
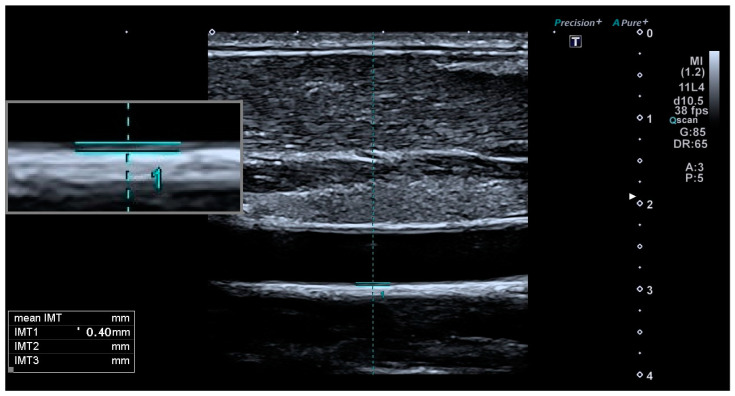
CIMT is measured as the distance between the lumen–intima and media–adventitia interfaces. This patient is from our control group; CIMT = 0.4 mm (normal).

**Figure 2:**
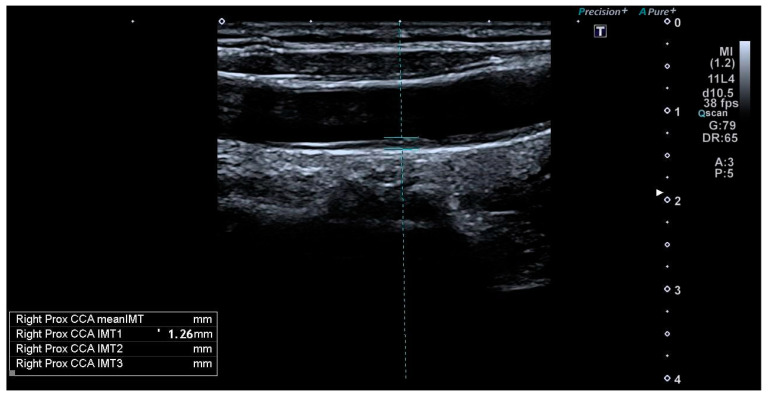
CIMT = 1.26 mm (distance between lumen–intima (line on top) and media–adventitia (line on the bottom) borders), measured in a 32-year-old transgender man who has been using testosterone for 7 years (considered pathological).

In situ (anatomical) and in vitro (histological) studies have confirmed that the two echogenic lines observed in ultrasound imaging of the distal wall of the carotid artery correspond to the lumen–intima unit and the media–adventitia interface [27]. The combined thickness of the inner two layers of the carotid artery wall, namely the intima and media layers, constitutes the CIMT. CIMT is measured as the distance between the boundaries of the lumen–intima and media–adventitia (Figure 1 and Figure 2) [28]. CIMT functions as a biomarker indicating the presence of early arterial wall thickening and potential underlying atherosclerotic changes. Consequently, elevated CIMT has been associated with early atherosclerosis and an increased risk of future cardiovascular events [29,30].

One of the primary challenges encountered in interpreting CIMT is the use of diverse measurement methods. Variations in measurement techniques can result in discrepancies and inconsistencies in CIMT values, making it difficult to compare results across studies and establish standardized thresholds for clinical interpretation. These methodological differences encompass variations in the examined carotid segment, the use of mean or maximum CIMT values, measurement of the near-and-far-wall or far-wall-only CIMT, single- or multiple-angle measurements, manual or automatic measurement techniques, assessment of the presence or absence of carotid plaques, and the choice between unilateral or bilateral measurements. To address these concerns and establish uniformity in CIMT measurement, the standards for CIMT measurement have been developed [28].

However, establishing a definitive threshold for abnormal CIMT values remains controversial. The relationship between CIMT and cardiovascular risk is continuous, making setting a definitive cutoff for abnormal CIMT values inappropriate. The latest 2018 guidelines from the European Society of Cardiology (ESC) and the European Society of Hypertension (ESH) reaffirm a CIMT value greater than 0.9 mm as an indication of asymptomatic organ damage. However, it is essential to recognize that middle-aged and elderly patients may exhibit higher thresholds, indicating cardiovascular risk [30,31]. Therefore, for accurate interpretation and clinical decision making based on CIMT values, individualized assessment and consideration of multiple risk factors are imperative.

In summary, CIMT serves as a surrogate marker of subclinical atherosclerosis, and CIMT screening enables clinicians to reclassify a significant proportion of patients from moderate cardiovascular risk into lower- or higher-risk categories [29]. In the context of transgender men, there is a paucity of studies focusing on cardiovascular markers within this population. The objective of this study was to examine the cardiovascular risks associated with long-term testosterone use in transgender men and assess CIMT as a potential novel biomarker for evaluating cardiovascular health in this specific population. By investigating the potential impact of testosterone hormone therapy on cardiovascular health in this unique population, the study aimed to contribute to our understanding of the cardiovascular effects of hormone therapy in transgender individuals.

## 2. Materials and Methods

This cross-sectional study was conducted at Istanbul University-Cerrahpasa (IUC), Cerrahpasa School of Medicine, Istanbul, between March 2022 and October 2022. The study was performed after obtaining the ethics committee approval E-83045809-604.01.01-402937 from the IUC Clinical Research Committee.

The study group consisted of 44 transgender men who had undergone hysterectomy and bilateral salpingo-oophorectomy (HBSO) at our institution between 2016 and 2023 and had been on exogenous testosterone hormone for over two years. The control group included 44 cisgender women individuals of similar age. The detailed inclusion criteria for the study and control groups are provided in Table 1.

Data collection included information on age, weight, sex and gender, smoking status, alcohol use, medical records, duration of menopause, exercise habits (self-reported), systolic and diastolic blood pressure, fasting glucose level, insulin level, HbA1c level, anti-Müllerian hormone (AMH) level, complete blood count, HDL, LDL, TG, and total cholesterol levels for both transgender men and cisgender women control subjects participating in the study with the aim to compare the metabolic and cardiovascular profiles between the two groups. HOMA-IR scores were calculated for all participants as a measurement of insulin resistance. This is a mathematical model that calculates systemic insulin resistance from insulin and glucose. Blood pressure measurements of all study participants were taken after a 5 min rest on the day of the radiological examination. In both groups, arterial blood pressure measurements were recorded from both upper extremities, with the highest measurement considered. 

On the days when blood samples were collected from both the study and control groups, carotid ultrasound imaging was conducted separately by two blinded radiologists, I.A. and O.A.K., with 24 and 5 years of ultrasound experience, respectively. They utilized a Canon Aplio i800 (Canon Medical Systems Inc., Tokyo, Japan) ultrasonography device equipped with an i18LX5 4–18 MHz linear probe. The investigators were unaware of the individuals’ identities and clinical results during the examination; although we took sufficient care about the blinding, this was realistically not possible because our groups were visibly identifiable. Additionally, only one investigator was present in the room at any given time. All patients and controls were examined in the supine position with slight neck hyperextension. Measurements of internal carotid artery intima–media (internal CIMT) and common carotid artery intima–media (common CIMT) thickness were conducted in the longitudinal section, with an insonation angle of 90 degrees to the vessel wall. Zoom settings were adjusted to enable the investigators to obtain the most accurate manual measurements. Measurements were recorded independently for both the right and left sides. To standardize the measurements, the reference point used was the carotid bifurcation. The measurement of the intima–media thickness (IMT) in the common carotid artery was taken 1 cm proximal to the reference point, while the measurement of the internal carotid artery was made 1 cm distal to the reference point. CIMT was manually measured as the distance between the lumen–intima and media–adventitia interfaces. All measurements were obtained from the far wall of the carotid artery, with segments containing atherosclerotic plaques excluded from the measurements. The thickness measurements from both the left and right sides, as determined by two investigators, were averaged for each subject. To assess the intra-observer reproducibility of CIMT measurements, each investigator repeated the same measurements in 20 randomly selected study subjects on a different day.

Given that the study group lacked pre-treatment CIMT values, the CIMT values of the transgender men in the study group were compared to those of the control group, which consisted of cisgender women without any chronic diseases.

### Statistical Analysis

In this study, statistical analysis was conducted using IBM SPSS Statistics version 20 (Statistical Package for the Social Sciences). The distribution of numerical values was evaluated using the Kolmogorov–Smirnov test. Student’s *t*-test was employed for normally distributed parametric values, while the Mann–Whitney U test was used for non-parametric values that did not exhibit a normal distribution, in order to assess the differences between the groups. The chi-square test was utilized to compare categorical values between the groups. Comparison of the parametric variables of the study group before and normally distributed parametric values are presented as mean ± standard deviation (mean ± SD), while non-parametric values without normal distribution are expressed as median (minimum value—maximum value). The intra and inter-observer agreement for the measurements between the two investigators was assessed using the intraclass correlation coefficient (ICC) with a 95% confidence interval. The ICC values for the thickness measurements exceeded 0.80, indicating a good agreement. The relationship between internal CIMT and common CIMT measurement values with other variables was examined using Pearson’s correlation analysis. All analyses were performed at a 95% confidence interval in this study, and a *p*-value < 0.05 was considered statistically significant. In addition, a power analysis was performed (power = 0.80, a = 0.05).

## 3. Results

A total of 88 individuals participated in the study, with 44 individuals in the study group comprising transgender men and 44 individuals in the control group comprising cisgender women. Within the study group, 11 patients were on testosterone undecanoate, while 33 patients were receiving testosterone isocaproate. The dosage and route of administration were consistent, with each individual receiving 250 mg intramuscularly. The duration of androgen administration ranged from 24 to 108 months, with a mean duration of 60.16 months and a median duration of 55 months. Comparison of demographic, clinical, and laboratory values of the study and control groups are provided in Table 2. The study group exhibited statistically higher mean body weight, systolic and diastolic blood pressure, hemoglobin, hematocrit, LDL, TG, and HbA1c levels than the cisgender control group. Conversely, the control group exhibited higher HDL levels. Moreover, the mean values of internal CIMT and common CIMT were higher in the study group, along with a greater percentage of individuals in this group having common CIMT values above the 75th percentile for their respective age and gender categories, as determined by the percentile charts provided the American Society of Echocardiography (ASE). Notably, there were no statistically significant differences between the groups regarding age, BMI, total cholesterol level, fasting blood glucose level, insulin level, and HOMA-IR values.

In our study, we measured the mean body weight, BMI, HDL, LDL, TG, and total cholesterol values of the transgender men before the initiation of gender-affirming hormone therapy and during the study period. Over the course of testosterone treatment, the study group showed increases in body weight, BMI, LDL, and TG values (Table 3).

To investigate the potential association between internal CIMT thickness and the cardiovascular risk status of transgender men, the correlation between internal CIMT thickness and various demographic, clinical, and laboratory values was examined. The results indicated that internal CIMT value was significantly correlated with common CIMT thickness (r1 = 0.738; p1 = 0.001), duration of treatment (r2 = 0.596; p2 = 0.003), systolic blood pressure (r3 = 0.274; p3 = 0.04), diastolic blood pressure (r4 = 0.248; p4 = 0.013), HDL level (r5 = 0.699; p5 = 0.001), and total cholesterol value (r6 = 0.389; p6 = 0.028).

Similarly, the correlation between common CIMT measurement values and various demographic, clinical, and laboratory values was also evaluated. The results showed that common CIMT was associated with internal CIMT (r1 = 0.722; p1 = 0.001), duration of treatment (r2 = 0.609; p2 = 0.001), duration of menopause (r3 = 0.402; p3 = 0.044), systolic blood pressure (r4 = 0.342; p4 = 0.005), and diastolic blood pressure (r5 = 0.705; p5 = 0.002).

When examining the effect of the duration of testosterone use in transgender men on the lipid profile and hemogram, a statistically significant positive correlation was observed between the duration of hormone therapy and hemoglobin, hematocrit, and TG values (r1 = 0.597, p1 = 0.001, r2 = 0.604, p2 = 0.001, r3 = 0.602, and p3 = 0.013, respectively). Additionally, a statistically significant negative correlation was found between the duration of drug use and HDL level (r = −0.028; *p* = 0.002).

## 4. Discussion

We conducted a study to investigate whether there is an increased risk of cardiovascular disease in transgender men who had been receiving testosterone for a prolonged time and had undergone early menopause due to surgery. CIMT is widely used as a predictor of atherosclerosis. We measured both conventional cardiovascular risk factors and CIMT values in transgender men with no other known risk factors and compared these measurements to those of cisgender women controls. We found that both internal and common CIMT values were significantly higher in the study group compared to the control group. Additionally, among the conventional cardiovascular risk factors assessed in the study group, we observed significant increases in body weight, BMI, LDL, TG, and total cholesterol levels as well as a significant decrease in HDL levels of the study group as compared to the control group.

The number of individuals undergoing medical and surgical gender transition is on rise. Studies indicate that individuals with gender dysphoria typically decide to transition during adolescence, and hormone replacement therapy is often initiated during this stage to develop male secondary sex characteristics [32]. However, some potential side effects are associated with using exogenous testosterone hormone in transgender men. Sex steroids such as testosterone and estrogen play a significant role in determining regional fat distribution. Numerous studies have reported increased BMI among individuals undergoing hormone therapy [2,4,8]. A recent meta-analysis examining the effects of testosterone on body weight and BMI revealed that transgender men experienced an increase in total body weight and lean body mass, along with a decrease in body fat [3]. In our study, we observed a similar trend, with an increase in body weight and BMI among the 44 transgender men following long-term testosterone therapy.

Transgender men experience early menopause due to HBSO surgery, which leads to the cessation of endogenous estrogen production [19]. The loss of the cardioprotective effects of estrogen at an early age increases the risk of atherosclerosis and cardiovascular disease [3,4,11,12,33]. A meta-analysis conducted by Elamin et al. investigated the effects of testosterone on cardiovascular disease risk factors, blood pressure, and lipid profile. The analysis revealed a direct correlation between the duration of testosterone use and a decrease in serum HDL levels as well as an increase in serum LDL, TG, and total cholesterol levels [18]. Additionally, testosterone hormone was found to significantly increase hemoglobin and hematocrit levels, leading to erythrocytosis [18]. In our study, we observed similar findings, including increased LDL and triglyceride levels and decreased HDL levels in the study group. Moreover, the study group exhibited significantly higher systolic and diastolic blood pressure levels and elevated Hb and Hct levels than the control group. However, it is important to note that various factors influence blood pressure values, and the higher blood pressure observed in our study may be attributed to unidentified genetic and environmental factors leading to endothelial damage. These factors were not accounted for in the demographic and clinical features considered.

Recently, carotid artery intima–media thickness (CIMT) measurement has gained popularity as a tool for assessing cardiovascular disease risk status due to its non-invasive nature, reliability, reproducibility, and cost effectiveness [26,28,29,30,34]. Numerous studies have demonstrated elevated CIMT values in patients with cardiovascular disease associated with atherosclerosis [26,29,30]. In our study, we compared the internal and common CIMT values between the study group of transgender men receiving long-term testosterone therapy and the control group of cisgender women. We observed higher internal CIMT and common CIMT values in the study group. Notably, there are only two studies evaluating CIMT in transgender men [35,36], and both studies found significantly higher CIMT values in transgender men: one over a baseline follow-up and another over cisgender women with PCOS. Our study fills a gap here as we compare transgender men who were under long-term gender-affirming hormone therapy to cisgender women. Our findings are consistent with these studies, and we additionally observed a positive correlation with testosterone exposure duration.

Determining the threshold for abnormal CIMT values remains a subject of debate. While the European Society of Cardiology (ESC) and European Society of Hypertension (ESH) suggest a CIMT value of >0.9 mm as pathological, the American Society of Echocardiography (ASE) defines a CIMT percentile value of >75th percentile, based on age and sex, as high and indicative of increased cardiovascular disease risk. In our study, we classified common CIMT values above the 75th percentile as elevated based on the percentile charts provided by the ASE [26]. This cutoff was used to define higher-than-normal common CIMT values in our analysis. Among the study group (n = 44), 64% (n = 28/44) had higher-than-normal common CIMT values compared to 16% (n = 7/44) in the control group (n = 44). Furthermore, we observed a positive correlation between the duration of testosterone use and internal and common CIMT values in the study group (r1 = 0.613, p1 = 0.002, r2 = 0.602, and p2 = 0.001).

Our study has certain limitations that should be acknowledged. Firstly, the small sample size of the study groups may have limited the statistical power and generalizability of the findings. Additionally, as a cross-sectional study, it does not assess the longitudinal changes in cardiovascular risk factors throughout the follow-up and treatment process of transgender individuals. Therefore, we cannot determine whether our findings justify more aggressive preventive therapy compared to standard care. Future studies with larger sample sizes and longitudinal designs are needed to investigate further the cardiovascular risks and optimal management strategies for transgender individuals.

## 5. Conclusions

The long-term outcomes and side effects of medical and surgical treatments in individuals with gender dysphoria have been inadequately studied. Identifying asymptomatic patients at high cardiovascular risk is important to prevent morbidity and mortality due to cardiovascular complications [17,18]. Given that testosterone hormone replacement therapy often begins in early adolescence and continues throughout life, and considering the potential negative effects of early menopause due to surgery, it is imperative to closely monitor these patients clinically and radiologically. This monitoring is essential to assess their cardiovascular disease risk and prevent long-term adverse health outcomes.

## Figures and Tables

**Table 1:** The inclusion criteria for both the study group (transgender men) and the control group (cisgender women) in the research study.

Study Group Inclusion Criteria
Transgender men between the ages of 20–50
Taking exogenous testosterone for more than 2 years
Undergone hysterectomy and bilateral salpingo-oophorectomy (HBSO)
No systemic diseases such as hypertension or diabetes mellitus
BMI < 30 kg/m^2^
No family history of coronary artery disease or peripheral arterial disease in first-degree relatives
No history of familial dyslipidemia
HDL, LDL, TG, total cholesterol, and hemogram results recorded prior to initiating gender-affirming hormone therapy
**Control Group Inclusion Criteria**
Cisgender women between the ages of 20–50
Not diagnosed with polycystic ovary syndrome
No systemic diseases such as hypertension or diabetes mellitus
BMI < 30 kg/m^2^
No family history of coronary artery disease or peripheral arterial disease in first-degree relatives
No history of familial dyslipidemia

**Table 2:** Comparison of demographic, clinical, and laboratory values of the study and control groups.

	Study Group (*n* = 44)	Control Group (*n* = 44)	*p*-Values
Age (years)	32.56 ± 6.479	36.8 ± 3.2	0.115
Body Weight (kg)	68.5 ± 11.2	60.8 ± 8.35	0.023 *
BMI (kg/m^2^)	25.3 ± 3.82	22.3 ± 4.1	0.096
Systolic Blood Pressure (mm/Hg)	117.6 ± 8.2	112 ± 7.2	0.013 *
Diastolic Blood Pressure (mm/Hg)	73 ± 5.9	66 ± 4.8	0.003 *
Hemoglobin (g/dL)	14.4 ± 1.03	12.5 ± 1.31	0.002 *
Hematocrit (%)	43.2 ± 4.1	37.5 ± 3.91	0.003 *
HDL (mg/dL)	49.9 ± 12.2	65.3 ± 24.1	0.042 *
LDL (mg/dL)	135.25 ± 32.12	115.43 ± 35.2	0.023 *
Triglyceride (mg/dL)	134.21 ± 52.52	75.5 ± 24.5	0.011 *
Total Cholesterol (mg/dL)	175.38 ± 46.1	195.83 ± 64.2	0.845
HbA1c (%)	5.72 ± 0.36	5.14 ± 0.21	0.006 *
Fasting Blood Glucose (mg/dL)	85.7 ± 7.2	78.6 ± 10.3	0.489
Insulin	8.6 ± 5.2	7.3 ± 3.2	0.454
HOMA-IR	1.92 ± 1.5	1.35 ± 0.72	0.346
Internal-CIMT (mm)	0.7 ± 0.13	0.59 ± 0.15	0.021 *
Common-CIMT (mm)	0.65 ± 0.15	0.56 ± 0.11	0.002 *
Higher-than-normal (>75th percentile **) Common-CIMT values (n/%)	28/44, %64	7/44, %16	0.003 *

*: *p* < 0.05 statistically significant. **: Based on the percentile charts provided by the American Society of Echocardiography (ASE).

**Table 3:** Comparison of clinical and laboratory values of the study group before treatment and during examination.

	Last Value (*n* = 44)	Initial Value (*n* = 44)	*p*-Values
Weight (kg)	68.5 ± 11.2	61.6 ± 9.4	0.001 *
BMI (kg/m^2^)	25.3 ± 3.82	21.5 ± 3.5	0.001 *
HDL (mg/dL)	49.9 ± 12.2	66.4 ± 22.6	0.001 *
LDL (mg/dL)	135.25 ± 32.12	110.46 ± 32.5	0.001 *
TG (mg/dL)	134.21 ± 52.52	72.4 ± 22.3	0.013 *
Total Cholesterol	211.992 ± 54.82	191.34 ± 59.56	0.388

* *p* < 0.05 statistically significant.

## Data Availability

Data is available upon reasonable request.
